# snpTree - a web-server to identify and construct SNP trees from whole genome sequence data

**DOI:** 10.1186/1471-2164-13-S7-S6

**Published:** 2012-12-07

**Authors:** Pimlapas Leekitcharoenphon, Rolf S Kaas, Martin Christen Frølund Thomsen, Carsten Friis, Simon Rasmussen, Frank M Aarestrup

**Affiliations:** 1 National Food Institute, Building 204, Technical University of Denmark, 2800 Kgs Lyngby, Denmark 4444; 2 Center for Biological Sequence Analysis, Building 208, Department of Systems Biology, Technical University of Denmark, 2800 Kgs Lyngby, Denmark

**Keywords:** whole genome sequencing (WGS), infectious disease epidemiology, single nucletide polymorphisms (SNPs), fastq

## Abstract

**Background:**

The advances and decreasing economical cost of whole genome sequencing (WGS), will soon make this technology available for routine infectious disease epidemiology. In epidemiological studies, outbreak isolates have very little diversity and require extensive genomic analysis to differentiate and classify isolates. One of the successfully and broadly used methods is analysis of single nucletide polymorphisms (SNPs). Currently, there are different tools and methods to identify SNPs including various options and cut-off values. Furthermore, all current methods require bioinformatic skills. Thus, we lack a standard and simple automatic tool to determine SNPs and construct phylogenetic tree from WGS data.

**Results:**

Here we introduce snpTree, a server for online-automatic SNPs analysis. This tool is composed of different SNPs analysis suites, perl and python scripts. snpTree can identify SNPs and construct phylogenetic trees from WGS as well as from assembled genomes or contigs. WGS data in fastq format are aligned to reference genomes by BWA while contigs in fasta format are processed by Nucmer. SNPs are concatenated based on position on reference genome and a tree is constructed from concatenated SNPs using FastTree and a perl script. The online server was implemented by HTML, Java and python script.

The server was evaluated using four published bacterial WGS data sets (*V. cholerae*, *S. aureus *CC398, *S*. Typhimurium and *M. tuberculosis*). The evalution results for the first three cases was consistent and concordant for both raw reads and assembled genomes. In the latter case the original publication involved extensive filtering of SNPs, which could not be repeated using snpTree.

**Conclusions:**

The snpTree server is an easy to use option for rapid standardised and automatic SNP analysis in epidemiological studies also for users with limited bioinformatic experience. The web server is freely accessible at http://www.cbs.dtu.dk/services/snpTree-1.0/.

## Background

The dramatic decrease in cost for whole-genome sequencing (WGS) has made this technology economically feasible as a routine tool for scientific research, including infectious disease epidemiology. In addition, WGS has major applications for health service providers working with infectious diseases [1] as such to deliver high-resolution genomic epidemiology as the ultimate typing method for bacteria.

The ideal microbial typing technique should enable differentiation of epidemiological unrelated strains and group epidemiological related (outbreak) strains, [2] and give information that will help to understand the evolutionary history of multiple strains within a clonal lineage [1,2]. Although some current technologies are highly informative like MLST or PFGE, they have limited resolution when applied to closely related isolates and different methods often have to be applied in different situations [1,2].

Especially outbreak isolates normally have very little diversity and require extensive genomic methods to differentiate and catagorize the isolates [3]. Single nucleotide polymorphisms (SNPs) also show relatively low mutation rates and are evolutionarily stable. Moreover, SNPs analysis has successfully been used for determining broad patterns of evolution in many recent studies [4-6].

Currently, There are a number of available non-commercial NGS genotype analysis software such as SOAP2 [7], GATK [8] and SAMtools [9]. Nonetheless, all of the software require bioinformatic skills, various options, various setting and they do not have a user friendly web-interface.

Here we introduce snpTree. A server for online-automatic SNP analysis and SNP tree construction from sequencing reads as well as from assembled genomes or contigs. The server is a pipeline which intregrates avaliable SNPs analysis softwares such as SAMtools [9] and MUMmer [10], with customized scripts. The performance of the server was evaluated with four published bacterial WGS data set; *Vibrio cholerae *[3], *Staphylococcus aureus *CC398 [6], *Salmonella *Typhimurium [11] and *Mycobacterium tuberculosis *[12].

## Implementation

**Figure 1:**
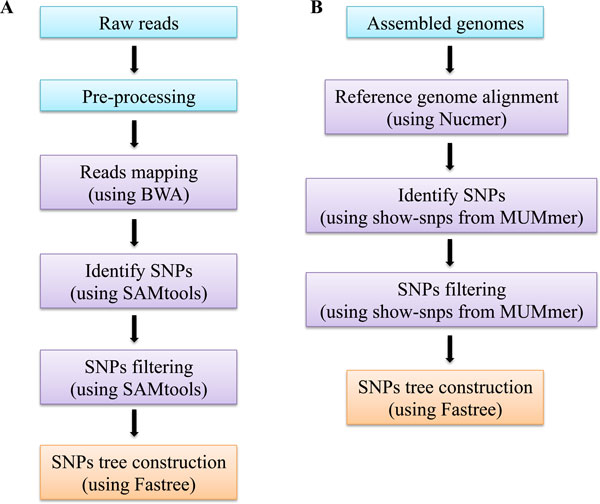
**snpTree server implementation**. (A) SNP tree construction from raw reads. Pre-processing (shown in blue) filters and trims raw data to remove low-quality bases. Trimmed raw reads are aligned against a reference genome by BWA with mapping quality equal to 30 as a default. SNPs calling and filtering process (shown in purple) identifies and filters informative SNPs by SAMtools with a couple of cut-offs, minimum coverage and minimum distance between each SNP (the default for both cut-offs is 10) and additionally all heterozygote SNPs are filtered. SNPs tree construction step (shown in orange) transforms from multiple alignments of concatenated SNPs to a phylogenetic tree by using Fastree and a perl script. (B) SNP tree construction from assembled genomes. Contigs or assembled genome are aligned to a reference genome using Nucmer. The SNPs calling and SNPs filtering steps are performed by a 'show-snps' application from MUMmer. SNPs tree construction step is carried out as the same way as the raw reads.

The snpTree server was created to handle both WGS data and assembled genomes to generate a phylogenetic tree based on SNPs data. The overall process is shown in Figure 1. For raw reads (Figure 1A), snpTree use an in-house toolbox (Genobox) for mapping and genotyping which consists of avaliable programs for next-generation sequencing analysis such as Burrows-Wheeler Aligner, BWA [13] and software package for SNPs calling and genotyping, SAMtools [9]. The source code of Genebox is available at https://github.com/srcbs/GenoBox. For contigs or assembled genomes (Figure 1B), MUMmer [10] is used for both reference genome alignment and SNPs identification processes.

The web-server contains more than 2,000 completed reference genomes collected from NCBI Genome database (accessed on April 2012).

### SNPs identification from WGS

Prior to mapping raw reads to a proper reference genome, the sequence data in fastq format are filtered and trimmed according to the following criteria [14]: (i) reads with N's are removed, (ii) if a read matches a minimum of 25 nt of a sequencing primer/adaptor the reads are trimmed at the 5' coordinate of match, (iii) the 3' tail bases are trimmed if the quality score is less than 20, (iv) the minimum average quality of the read should be 20 and the read length after trimming should be at least 20 nt.

Trimmed raw reads are aligned against a reference genome using BWA [13] with minimum mapping quality equal to 30 as a default (Figure 1A). BWA is based on an effective data compression algorithm called Burrows-Wheeler transform (BWT) that is fast, memory-efficient and espectially useful for aligning short reads [15].

SNPs calling and filtering are accomplished by SAMtools that is a software package for parsing and manipulating alignments in the generic alignment format (SAM/BAM format) [9]. The snpTree server allows users to set a couple of parameters to filter SNPs, a minimum coverage and a minimum distance between each SNPs (prune). The default for both cut-offs is set to 10 and additionally all heterozygous SNPs are filtered because these are likely mapping errors in haploid chromosomes. The identifed SNPs are concluded into a VCF file.

### SNPs identification from assembled genomes

A pipeline has been developed around the software package MUMmer version 3.23 [10] (Figure 1B). An application named Nucmer, which is part of MUMmer, is used to align each of *de novo *assemblies to a reference genome chosen by the user (default settings). SNPs are then called from the resulting alignments with another MUMmer application named "show-snps" (with options "-CIlrT"). A pruning is then applied, if chosen by the user, and the SNPs are written into a VCF formatted file for each of the analyzed genomes.

### SNPs tree construction

One VCF formatted file is needed for each Operational Taxonomic Unit (OTU). The SNPs are then concatenated into a single alignment by ignoring indels. Including indels would disturb the position of SNPs in the sigle alignment. To include indels in any trees, it requires some sensible way to represent them numerically as distances in an evolutionary space, and there is no any ways to achieve this. Indels could theoretically be included in a multiple sequence alignment, since such alignments can handle gaps but it's difficult to score them. "Blast-like" gap penalties certainly would not work, since they are optimized for much larger gaps, e.g. recombination events.

It is important to note that SNPs not found in a VCF file is interpreted as not being a variation and the corresponding base in the reference is expected. This might not always be the right choice, because a SNP not found in a VCF file could be a result of an INDEL. It is expected to be a rare case and probably won't disturb the phylogenetic signal.

The alignment is passed on to Fastree [16], which creates a maximum likelihood tree from the SNP alignment.

### snpTree server output

**Figure 2:**
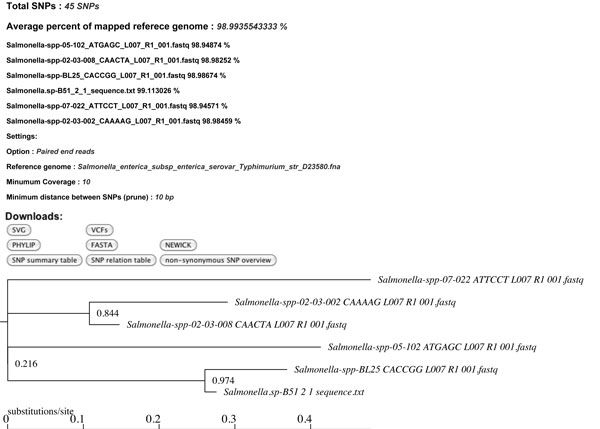
**snpTree output**. An example of the output from snpTree server using Illumina paired-end reads as input data.

snpTree server provides an output to users with SNPs tree figure in SVG format, number of SNPs and other relevant output files such as (i) SNPs files, which contains identified SNPs including indels for each input genome in VCF format [17], (ii) cancatenated SNPs in newick, phylip and fasta format, (iii) SNPs annotation files which give users an overview of nucleotide changes or amino acid changes from SNPs including which input genomes contain which SNPs as well as information about synonymous and non-synonymous SNPs (Additional file 1). An example of output is shown in Figure 2.

## Results and discussion

The snpTree was evaluated using raw reads and assembled genomes from four published bacterial WGS data sets (*V. cholerae *[3], *S. aureus *CC398 [6], *S*. Typhimurium [11] and *M. tuberculosis *[12]). The evaluation was considered based on tree topology as well as the reference genome's position of identifed SNPs.

### Evaluation of tree topology and SNPs position

WGS from published data set were subjected to snpTree server in order to generate SNP trees. The tree topology evaluation was based on percentage of concordance. If the strain in the tree from snpTree server matches exactly with the tree from published data, it was considered as an exact match. If the strains were grouped into the same cluster with published data, it was considered as a cluster match. In addition, the snpTree server was evaluated with assembled genomes or contigs. The raw reads were assembled prior by *de novo *assembly using Velvet 1.1.04 [18]. The assembled genomes were processed to snpTree server to make SNP trees.

### *V. cholerae *data set

**Figure 3:**
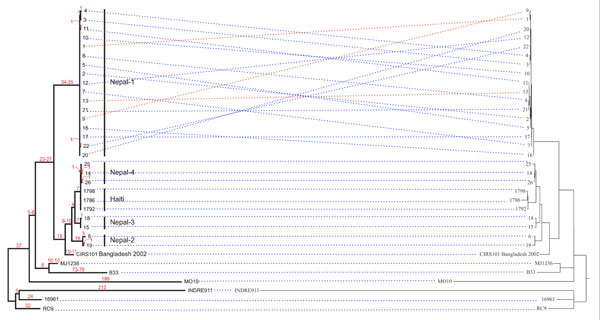
**Comparison between phylogenetic trees from published data set (*V. cholerae*) and snpTree server**. These trees (34 WGS from ***V. cholerae***) shows comparison of tree topology between the trees from original publication (left) and snpTree server (right). The linked lines indicate exact match for each genome in the tree. According to the tree from published data, the blue lines mean exact match and the red one represent inexact match.

The evaluation results are summarized in Table 1. For the *V. cholerae *data set, the performance of snpTree from raw reads (Figure 3) and contigs (Additional file 2) were accurate in term of exact match and cluster match. From Figure 3, all of genomes were grouped into the same clusters as in the original tree. In the Nepal-1 cluster, there are only 3 genomes that are not in the same position compared to the original tree. However, the isolates in Nepal-1 group are highly homogeneous and there are some synapomorphic SNPs (genome position that has mutated the new nucleotide which shared with all descendants) supporting its unique identities [3].

**Table 1:** Evaluation table

Data set	Percentage of concordance	
	
	Exact match	cluster match
*V. cholerae *(raw reads)	91	100
***V. cholerae *(contigs)**	**85**	**100**
*S. aureus *CC398 (raw reads)	88	96
***S. aureus *CC398 (contigs)**	**87**	**97**
*S*. *typhimurium *(raw reads)	61	100
***S*. *typhimurium *(contigs)**	**53**	**100**
*M. tuberculosis *(raw reads)	58	78
***M. tuberculosis *(contigs)**	**25**	**72**

The percentage of concordance from comparing SNP trees from snpTree server against the four published data set.

**Figure 4:**
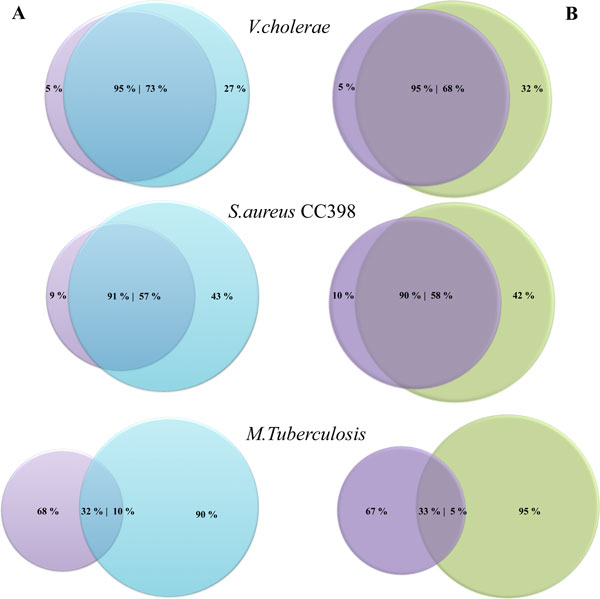
**Percentage of identified SNPs**. Venn diagram showing the percentage of overlapped and non-overlapped identified SNPs from snpTree server against original publications in both raw reads (A) and assembled genomes (B). The purple, blue and green circles represent the percentage of identified SNPs from original publications, raw reads and assemble genomes from snpTree server respectively.

The percentage of overlapped and non-overlapped SNPs between published data and snpTree server is illustrated in Figure 4A for raw reads and Figure 4B for assembled genomes. For *V. cholera*, both raw reads and contigs (Figure 4), the snpTree server identified SNPs mostly from the same position in published data (95% overlapped SNPs). This result supports the consistency of the tree from snpTree server (Figure 3).

### *S.aureus *CC398 data set

For *S. aureus *CC398 (Table 1), snpTree produced a tree with 87 - 88 % concordance for exact match and 96 - 97 % concordance for cluster match. SNP trees for raw reads and assembled genomes are shown in Additional file 3 and Additional file 4 respectively. There were 91 and 90 % overlapping SNPs for raw reads and assembled genomes (Figure 4). The performance of snpTree on this data set was slightly less than for the *V. cholera *data set. The reason is probably that the genomes of 89 *S. aureus *CC398 isolates came from animals and humans sources from 19 countries and four continents. In addition, there are 4,238 SNPs among them [6]. These isolates are more diverse than *V. cholera *isolates. Thus, this diversity makes difficulty for snpTree to capture exactly the same variant as in original publication. Nevertheless, snpTree can differentiate between isolates from humans and pigs which is very meaningful to epidemiological studies.

### *S*. Typhimurium data set

The third data set, *S*. Typhimurium, which consists of 51 *Salmonella *in which 43 isolates from 14 patients with multiple recurrences in Blantyre, Malawi and 8 control typhimurium isolates [11]. Like in the original publication, both raw reads and contigs data set, the isolates fell within three distint phylogenetic clusters (Additional file 5 and 6) which gave 100 % concordance for cluster match (Table 1). On the other hand, the percentage of concordance for exact match was quite low (53 - 61 %). It is not possible to evaluate SNPs position for this data set because of lacking SNPs position data. However, the number of identified SNPs from snpTree server (1,692 SNPs) was not much different from original data set (1,463 SNPs). Most of the *S*. Typhimurium isolates are highly genetically related as they came from patients who had recrudescence and/or reinfections. Therefore, this study requires high-resolution SNPs analysis and intensive phylogenetic tree construction to differentiate these little variation. In addition, the original tree from this data set was generated and confirmed using several independent approaches, with bootstrap support and clade credibility marked [11] which snpTree cannot repeat as using bootstrapping is time-consuming.

### *M.tuberculosis *data set

Another data set that consists of 32 *M. tuberculosis *outbreak isolates and 4 historical isolates (from the same region but isolated before the outbreak) with matching genotype suggesting that the outbreak was clonal [12]. The performance of snpTree server on this data set was inconsistent due to low concordance percentage for exact match and cluster match (Table 1, Additional file 7 and 8). Moreover, the number of indentified SNPs and matching SNP positions (Figure 3) are very different between the tree from snpTree server (677 SNPs) and the published data (204 SNPs). The original publication determined transmission dynamics of the outbreak at a higher resolution by filtering to remove many of SNPs in repetitive regions and those appearing in a single isolate. Thus, the procedure in the original manuscript is impossible to repeat and it should be noted that the original filtering reduced the number of SNP's from more than 1,000 to 204. This is probably the reason that snpTree were unable to reproduce the same results as in the original publication.

### Sensitivity and specificity

In order to evaluate the sensitivity and specificity of SNP calling method, the artificial sequence was created from a genome of 4,878,012 bp with 1,000 randomly SNP artificial inserted. The simulated sequence was aligned to a reference genome and identified SNPs using SNP idenfication pipeline for assemble genome. SNPs calling was performed with varied two cut-off values which are minimum number of bp between SNPs (prune) and minimum number of bp from a sequence end (e). The sensitivity and specificity for SNP identification were summarized in Table 2.

**Table 2:** Sensitivity and specificity

Variable and cut-off value	Sensitivity (%)	Specificity (%)
***Number of bp between SNPs***		
0	97.8	100
10	97.2	99.99988
25	96.6	99.99975
50	95.8	99.99959
75	94.6	99.99935
100	93.8	99.99918
		

***Number of bp from a sequence end***		

0	97.8	100
10	97.8	100
25	97.8	100
50	97.8	100
75	97.8	100
100	97.7	100

Evaluation of sensitivity (SN) and specificity (SP) using different settings of minimum number of bp between SNPs (prune) and minimum number of bp from a sequence end (e) for SNP detection on a simulated dataset consisting of a genome of 4,878,012 bp with 1,000 randomly SNP artificial inserted.

The sensitivity for prune cut-off (Table 2) was slightly dropped when increasing number of prune. This is due to the more number of bp between SNPs (prune) leading to the high chance to have SNPs between that number of bp.

Using minimum number of bp from a sequence end as a varied cut-off, the sensitivity was very high and stable for all varied values. It is quite rare to have SNPs occurred in the tails of sequence so this cut-off less affects to the SNP calling process. The specificity for both cut-off were very high. It is because the number of SNP inserted is extreamly low (1,000 SNPs) compared to the whole genome (4,878,012 bp).

The rapid technological advantages in WGS and rapidly decreasing cost has made the technology available for large groups of scientists as well as clinical microbiologists. It is expected that WGS will very soon find widespread use in clinical and public health microbiology, as has already been shown [19]. The implementation of such technologies will however, create a major need for simple to use bioinformatic tools to make sense of the data generated. We have here developed snpTree and evaluated it on four different published datasets. The concordance of the SNPs tree from raw reads was more adequate than the one from assembled genomes, which is not surprising. However, in practice transfering sequencing reads will be more time-consuming than just transferring assembled genomes and the tree topology from these different kind of genomes was only sligthly different. Therefore, the assembled genomes option in snpTree server can provide a quicker solution for uploading time-consuming. In order to create informative SNPs tree, using a closely related reference genome is important. Therefore, the selection of a proper reference genome is crucial. Thus, it is adviced to choose a reference genome belonging to the same or as closely related a sub-type as possible to the strain collection under study. This could for species where this is a available reference belonging to the same MLST type. In the future a more generic solution to overcome this obstracle might be to using high-resolution prediction method such as K-mers to assign a genuine reference genome.

## Conclusions

The advance of WGS and the use of epidemiological genomics underline the potential of practical application of WGS for clinical microbiology and emphazies the importance of biology and evolution in developing reliable and accurate genomics tools for clinical use. In addition, SNP-typing phylogenetic methods can distinguish very closely related isolates to a degree not achievable by widely employed sub-genomic typing tools. snpTree server might be not a perfect tool but it is an option for easy and rapid standardised and automatic SNP analysis tool in epidemiological studies. It is also useful for users with limited bioinformatic experience.

## Competing interests

The authors declare that they have no competing interests.

## Authors' contributions

PL planned the study, carried out web-server construction and drafted the manuscript. RKM constructed SNPs analysis pipeline for assembled genomes and automatic SNP tree construction pipeline. MCFT participated in web-server construction. CF constructed automatic SNPs tree construction pipeline. SR constructed SNPs analysis pipeline for raw reads and developed Genobox toolbox. FMA supervised, planned the study and drafted the manuscript. All authors have read and approved the final manuscript.

## Supplementary Material

Additional file 1 **Example of SNP annotation output**. Click here for file

Additional file 2 **SNP trees from contigs of *V. cholerae *data set (left is the tree from original publication and right is the tree from snpTree server)**. Click here for file

Additional file 3 **SNP trees from raw reads of *S. aureus *CC398 data set (left is the tree from original publication and right is the tree from snpTree server)**. Click here for file

Additional file 4 **SNP trees from contigs of *S. aureus *CC398 data set (left is the tree from original publication and right is the tree from snpTree server)**. Click here for file

Additional file 5 **SNP trees from raw reads of *S*. Typhimurium data set (left is the tree from original publication and right is the tree from snpTree server)**. Click here for file

Additional file 6 **SNP trees from contigs of *S*. Typhimurium data set (left is the tree from original publication and right is the tree from snpTree server)**. Click here for file

Additional file 7 **SNP trees from raw reads of *M. tuberculosis *data set (left is the tree from original publication and right is the tree from snpTree server)**. Click here for file

Additional file 8 **SNP trees from contigs of *M. tuberculosis *data set (left is the tree from original publication and right is the tree from snpTree server)**. Click here for file
