# Supplementary figures and images for: snpTree - a web-server to identify and construct SNP trees from whole genome sequence data
Source: BMC Genomics. 2012 Dec 7;13(Suppl 7):S6. doi: 10.1186/1471-2164-13-S7-S6 (PMC3521233; doi:10.1186/1471-2164-13-S7-S6)

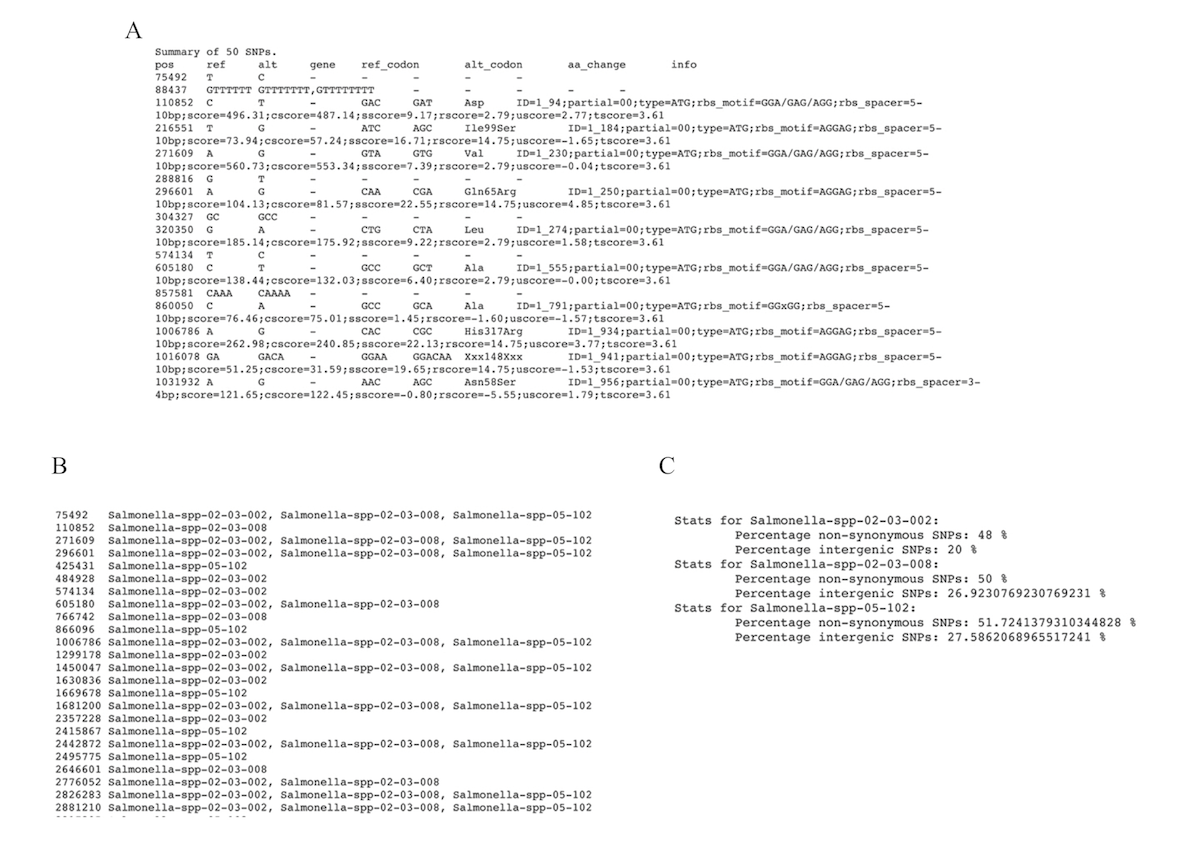

Supplement: Additional file 1 — Example of SNP annotation output. [file 1471-2164-13-S7-S6-S1.PNG]

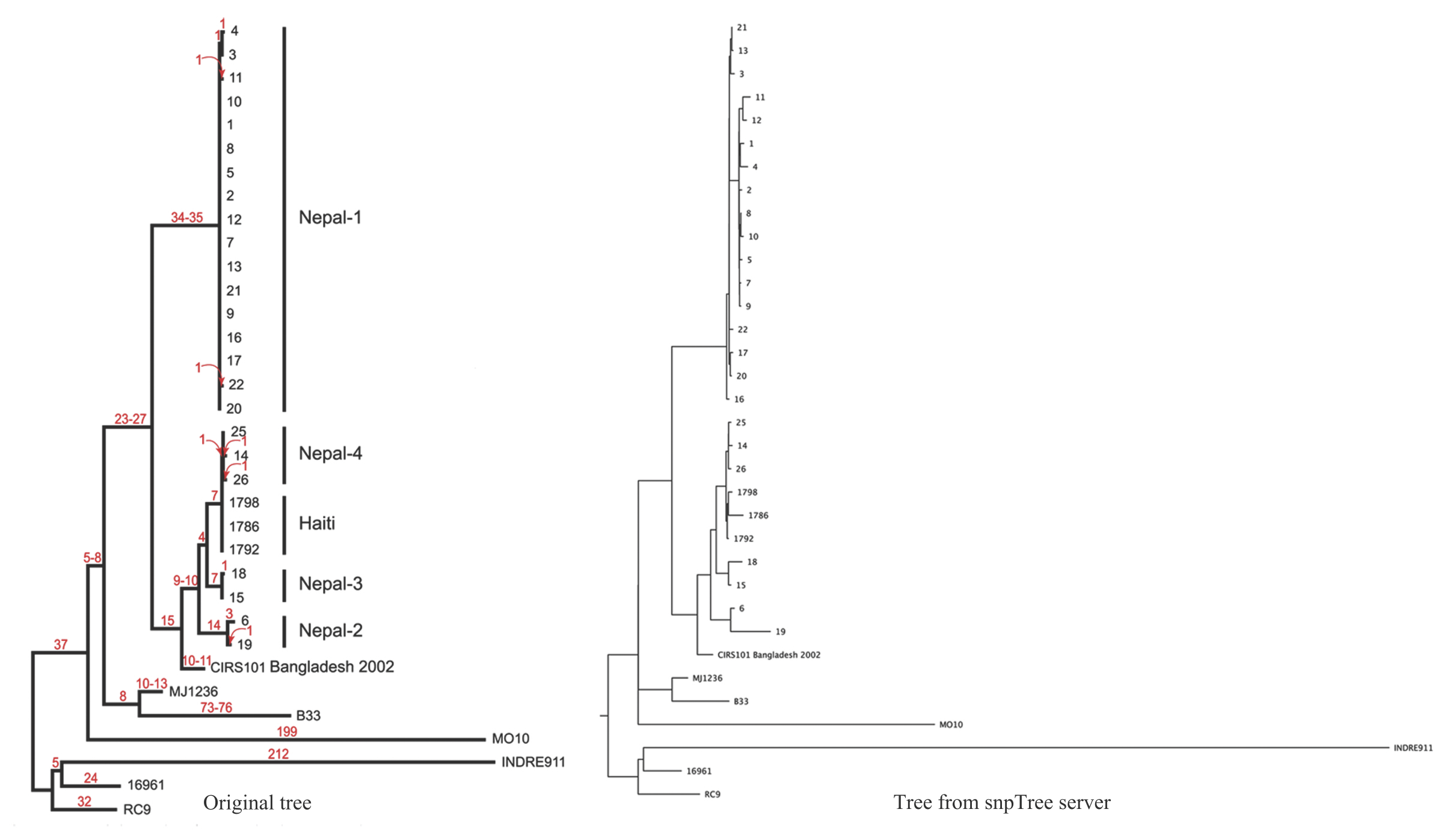

Supplement: Additional file 2 — SNP trees from contigs of V. cholerae data set (left is the tree from original publication and right is the tree from snpTree server). [file 1471-2164-13-S7-S6-S2.PNG]

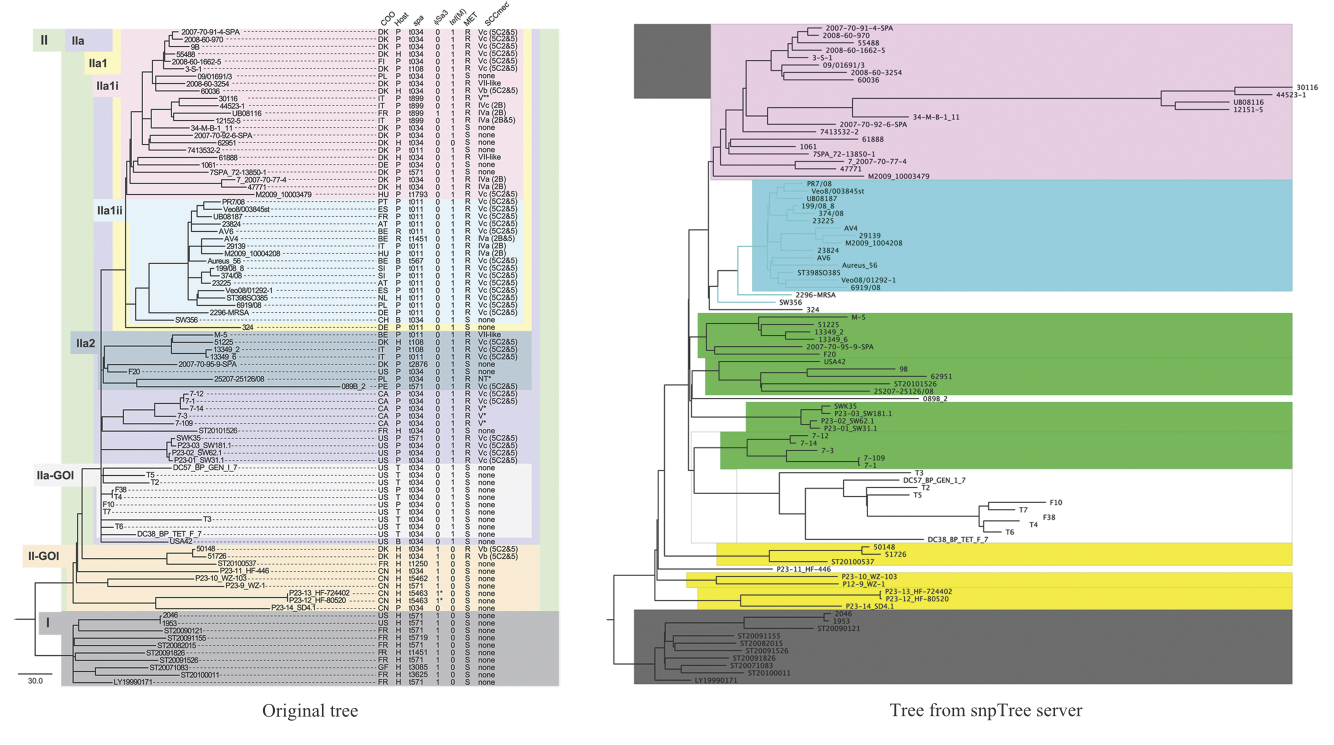

Supplement: Additional file 3 — SNP trees from raw reads of S. aureus CC398 data set (left is the tree from original publication and right is the tree from snpTree server). [file 1471-2164-13-S7-S6-S3.PNG]

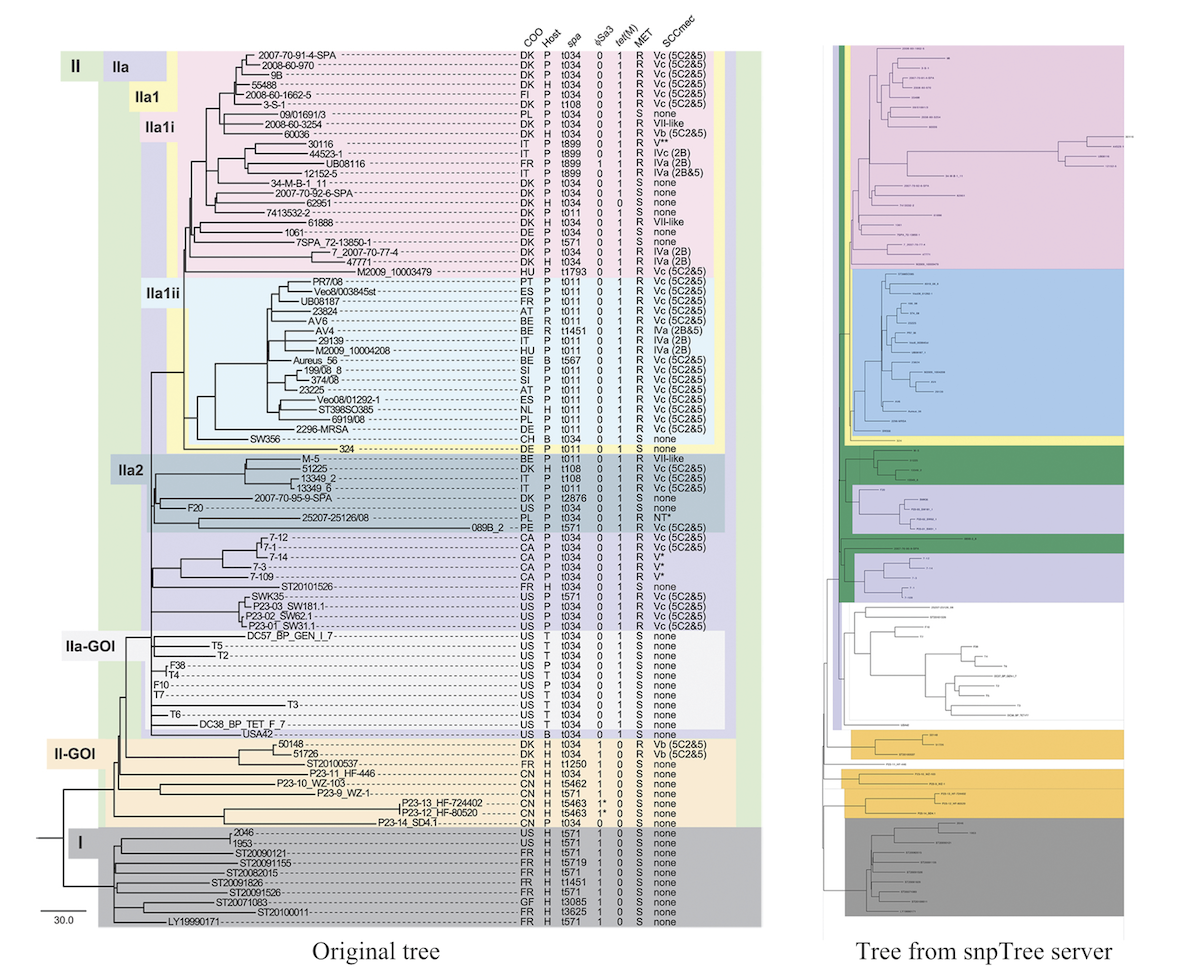

Supplement: Additional file 4 — SNP trees from contigs of S. aureus CC398 data set (left is the tree from original publication and right is the tree from snpTree server). [file 1471-2164-13-S7-S6-S4.PNG]

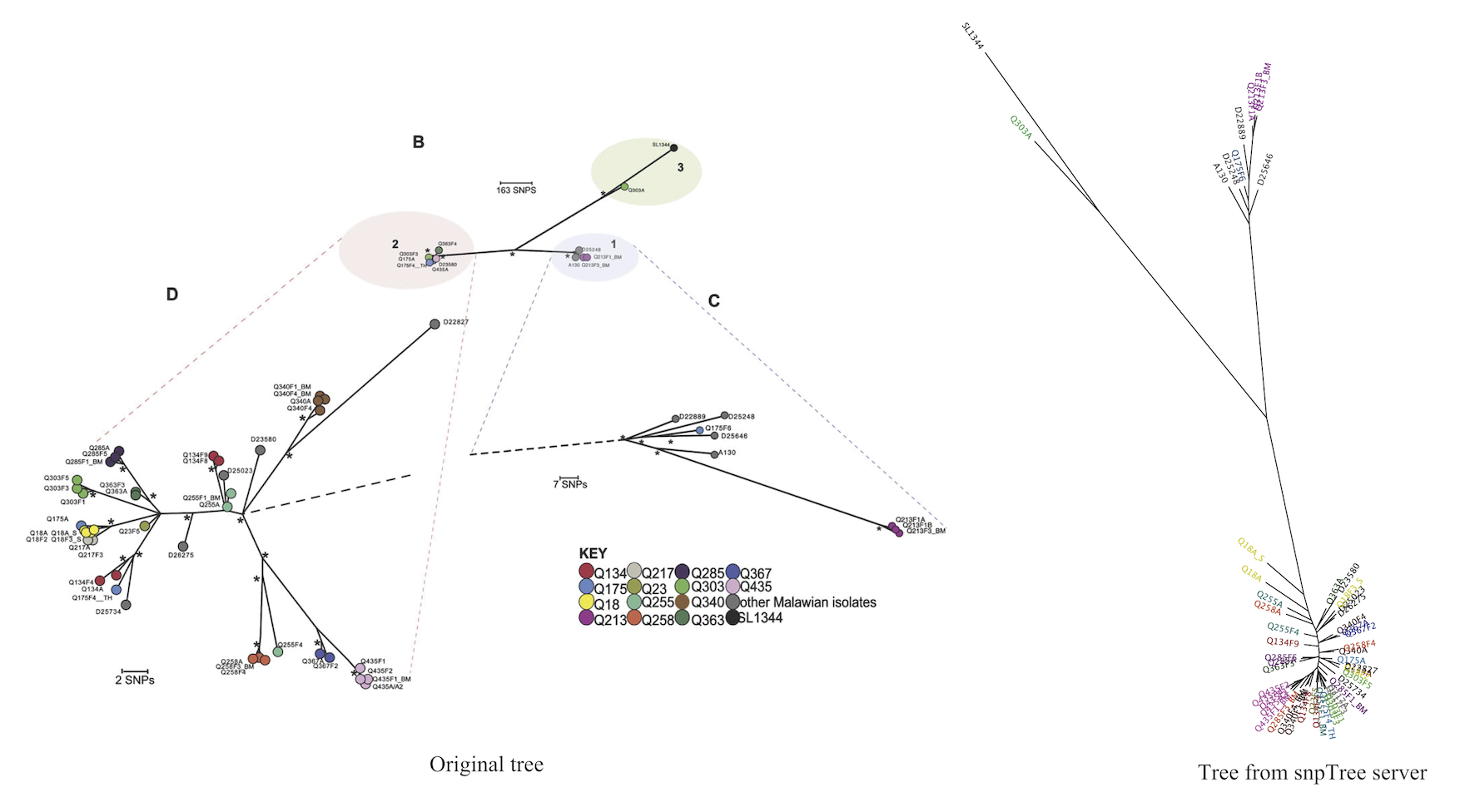

Supplement: Additional file 5 — SNP trees from raw reads of S. Typhimurium data set (left is the tree from original publication and right is the tree from snpTree server). [file 1471-2164-13-S7-S6-S5.PNG]

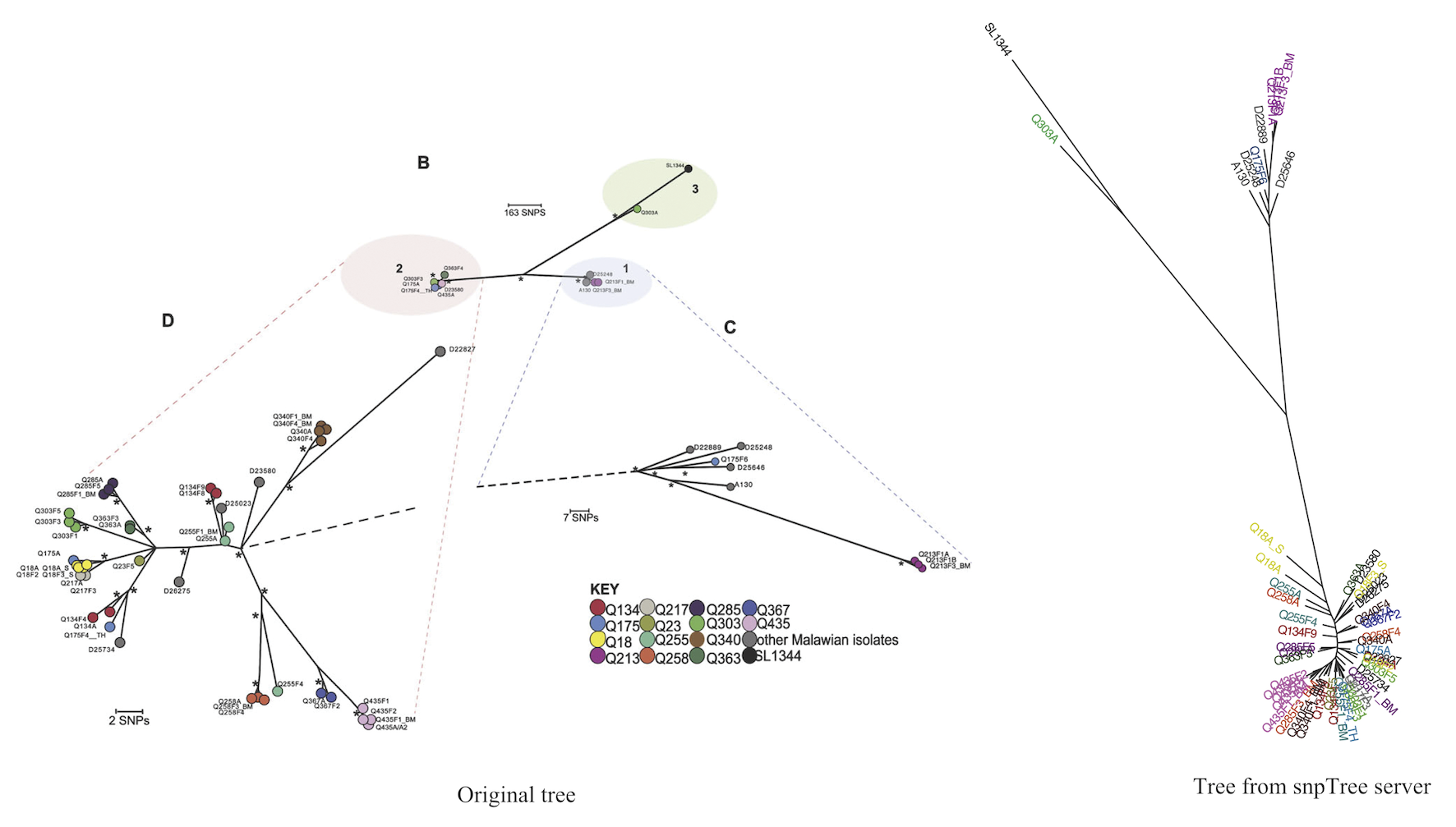

Supplement: Additional file 6 — SNP trees from contigs of S. Typhimurium data set (left is the tree from original publication and right is the tree from snpTree server). [file 1471-2164-13-S7-S6-S6.PNG]

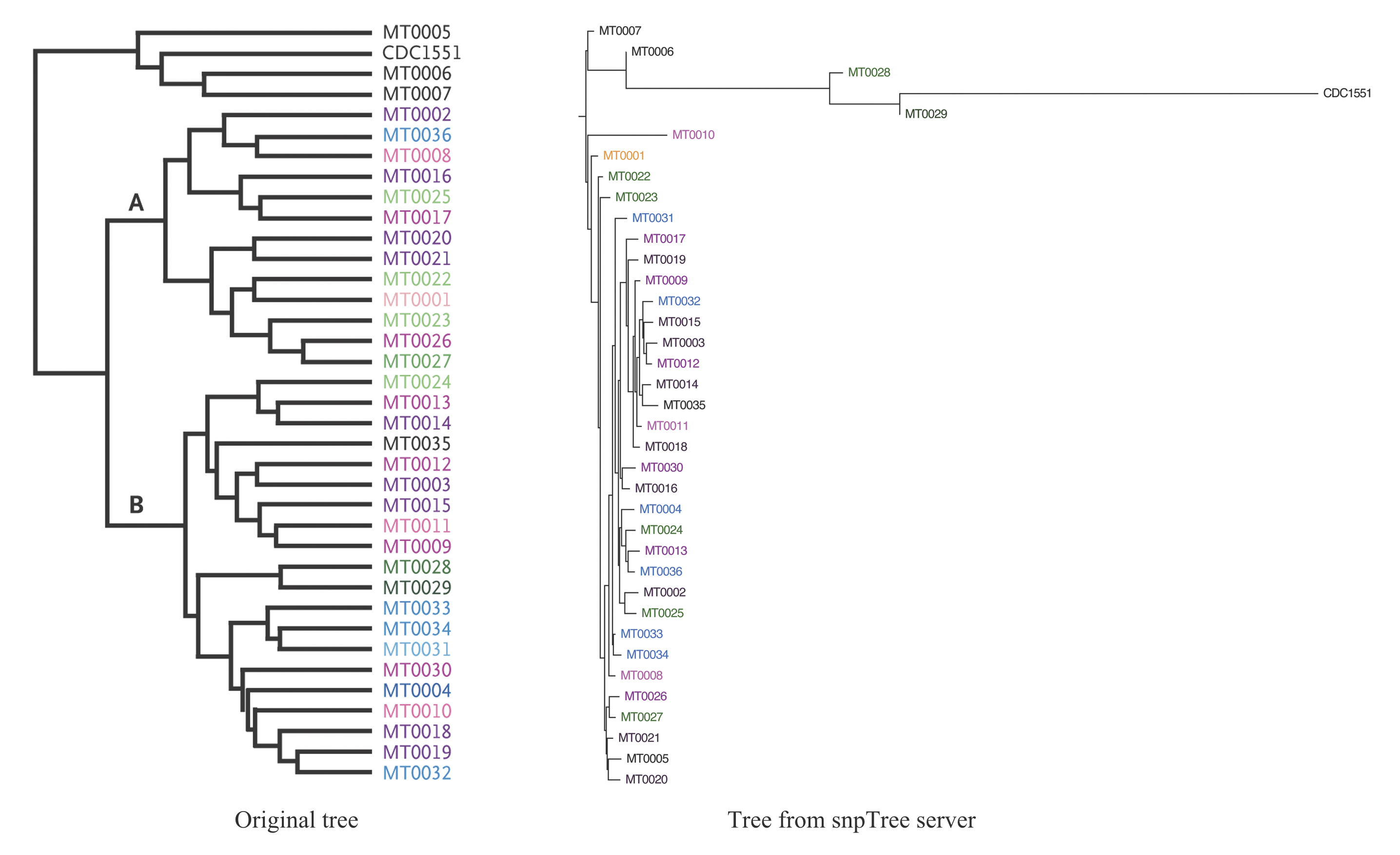

Supplement: Additional file 7 — SNP trees from raw reads of M. tuberculosis data set (left is the tree from original publication and right is the tree from snpTree server). [file 1471-2164-13-S7-S6-S7.PNG]

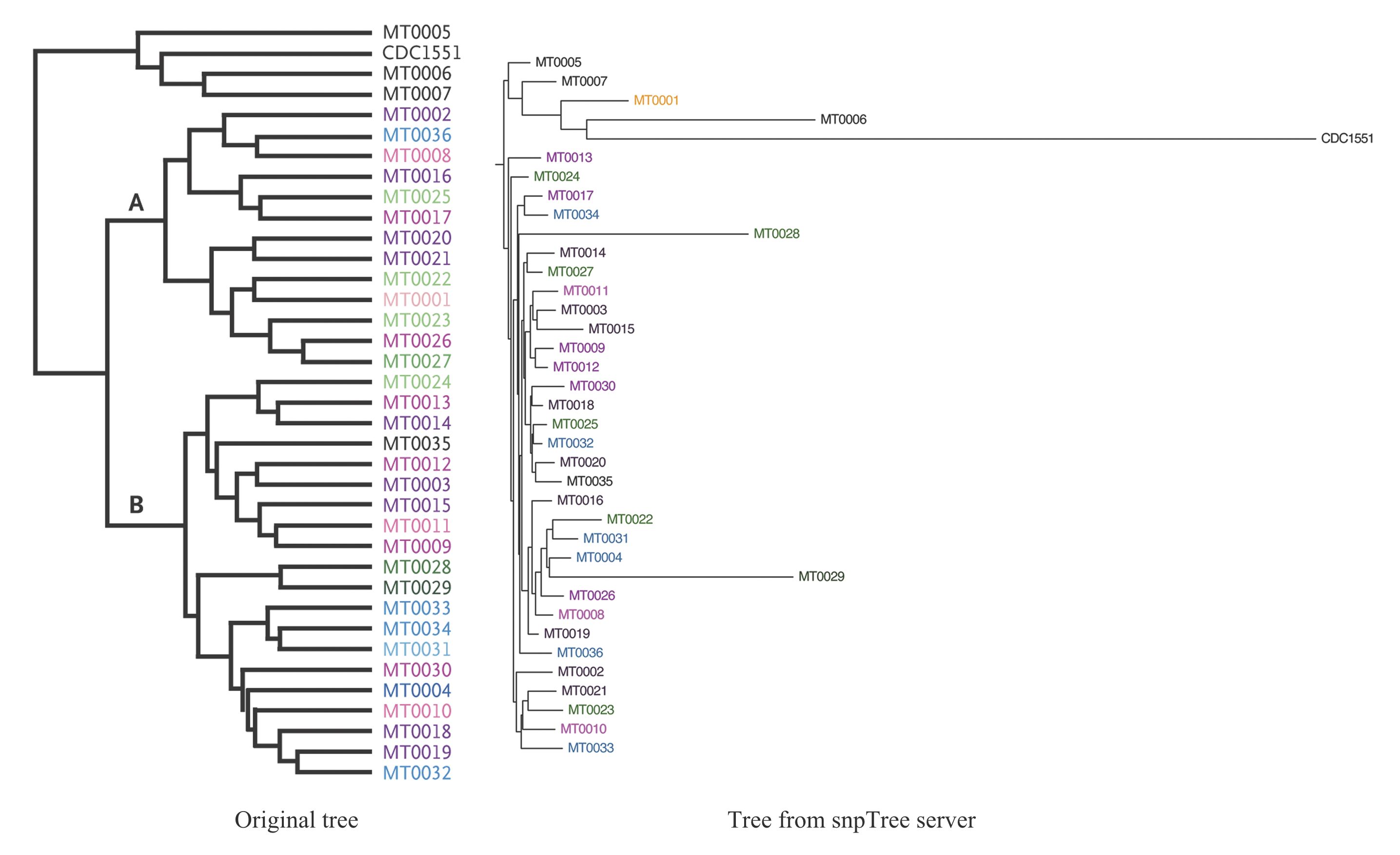

Supplement: Additional file 8 — SNP trees from contigs of M. tuberculosis data set (left is the tree from original publication and right is the tree from snpTree server). [file 1471-2164-13-S7-S6-S8.PNG]
